# THE LONGITUDINAL ASSOCIATIONS BETWEEN SOCIAL NETWORK STRUCTURE AND SELF-ESTEEM

**DOI:** 10.1093/geroni/igac059.149

**Published:** 2022-12-20

**Authors:** Rita Hu, Toni Antonucci

**Affiliations:** University of Michigan, Ann Arbor, Michigan, United States; University of Michigan, Ann Arbor, Michigan, United States

## Abstract

Based on the convoy model of social relations, the current study used Latent Growth Curve Modeling to examine the associations between overall network, closest, close and weak social tie trajectories across the lifespan and self-esteem later in life. Participants (N = 553) aged 13 to 77 in Wave 1 (1992) were surveyed again in 2005 (Wave 2) and 2015 (Wave 3). The overall network size increased significantly across the lifespan (β = 0.56, SE = 0.01, p < 0.001). The closet tie size trajectories were not significantly associated with self-esteem 23 years later. The growth of the close tie size was not significantly associated with self-esteem later. Weak-tie size growth was also significantly associated with higher self-esteem later (β = 0.14, SE = 0.00, p < 0.05). The findings highlight social network’s effects on self-esteem across the lifespan, as well as the critical role weak social ties play in development.

